# Human brucellosis: seroprevalence and associated exposure factors among abattoir workers in Abuja, Nigeria - 2011

**DOI:** 10.11604/pamj.2013.16.103.2143

**Published:** 2013-11-17

**Authors:** Mabel Kamweli Aworh, Emmanuel Okolocha, Jacob Kwaga, Folorunso Fasina, David Lazarus, Idris Suleman, Gabrielle Poggensee, Patrick Nguku, Peter Nsubuga

**Affiliations:** 1 Nigeria Field Epidemiology and Laboratory Training Programme (NFELTP), Abuja, Nigeria; 2 Department of Veterinary Public Health and Preventive Medicine, Ahmadu Bello University, Zaria, Nigeria; 3 Department of Production Animal Studies, Faculty of Veterinary Science, University of Pretoria, Onderstepoort 0110, South Africa and National Veterinary Research Institute, Vom, Nigeria; 4 Department of Community Medicine, Ahmadu Bello University, Zaria, Nigeria; 5 Global Public Health Solutions, Atlanta, USA

**Keywords:** Brucellosis, seroprevalence, exposure factors, abattoir, occupational hazard, Nigeria

## Abstract

**Introduction:**

Brucellosis, a neglected debilitating zoonosis, is a recognized occupational hazard with a high prevalence in developing countries. Transmission to humans can occur through contact with infected animals or animal products. Brucellosis presents with fever. In Nigeria, there is a possibility of missed diagnoses by physicians leading to a long debilitating illness. We conducted a study to determine the seroprevalence and factors associated with Human Brucellosis (HB) among abattoir-workers in Abuja, Nigeria.

**Methods:**

We conducted a cross-sectional study and selected abattoir-workers using stratified random sampling. Structured questionnaires were used to collect data on demographics and exposure-factors. We tested the workers’ serum-samples using Rose-Bengal (RBPT) and ELISA tests. A worker with HB was one whose serum tested positive to RBPT or ELISA. We tested differences in proportions between workers with HB and those without HB using odds-ratio and X^2^ tests.

**Results:**

Of 224 workers, 172 (76.8%) were male and mean age was 30 + 9.0 years. Of 224 sera collected, 54 were positive giving a seroprevalence of 24.1%. Of these, 32 (59.3%) were butchers, and 11 (20.4%) were meat-sellers. Slaughtering animals while having open-wounds (Odds-ratio (OR) = 2.15, 95% Confidence Interval (CI) = 1.15-4.04); occupational-exposure of >5years (OR = 2.30, CI = 1.11-4.78) and eating raw meat (OR = 2.75, CI = 1.21-6.26) were significantly associated with HB. Multivariate analyses showed that occupational-exposure of >5years (Adjusted OR (AOR) =2.45, CI = 1.15 – 5.30) and eating raw-meat (AOR = 2.64, CI = 1.14 - 6.14) remained significantly associated with HB.

**Conclusion:**

Seroprevalence of HB among abattoir-workers in Abuja was high. Factors associated with HB were occupational-exposure of >5years and eating raw-meat. Abattoir-workers should be discouraged from eating raw-meat and educated on adherence to safe animal-product handling practices.

## Introduction

Brucellosis, a zoonotic disease of public health importance and a neglected animal disease has been eradicated in many developed countries [1]. It however remains one of the seven zoonotic diseases listed by the World Health Organization (WHO) as “neglected”. The disease has a great impact on both animal and human health as well as tremendous socio-economic impact in developing countries where rural income relies largely on livestock breeding and dairy products [2]. Brucellosis is endemic in livestock in most countries of Africa including Nigeria [3, 4] and is an established endemic disease in slaughtered cattle population with a prevalence of 16.2% in sub-Saharan Africa (2002) [5] and 3.5% in Nigeria (2001) [6, 7].

Several species of Brucella that are important to public health exist amongst which *B melitensis* and *B suis* are more virulent for humans than *B abortus* and *B canis*although serious complications can occur with any species of Brucella [8]. Humans are infected either by direct contact with blood, placenta or uterine secretions of infected animals, through breaks in the skin, by inhalation or by ingestion of unpasteurized milk and other dairy products [9]. It is known that unpasteurized milk is sold in several parts of Nigeria [10]. Brucellosis is an occupational hazard to individuals engaged in certain professions such as abattoir workers, veterinarians, livestock farmers and herdsmen [11].

This study focuses on abattoir workers in two abattoirs in Abuja. This is because brucellosis is a recognized occupational hazard which is rarely diagnosed in most health facilities in the country, and is yet to be included in the Integrated Disease Surveillance and Response (IDSR) system - a public health surveillance and response system for priority diseases [12]. Very few studies have been done to assess the prevalence of brucellosis in abattoir workers in Nigeria. To our knowledge, there is no published literature on the brucellosis status of this occupational group in Abuja. In addition to this, the Federal Capital Territory (FCT) Department of Agriculture and Rural Development is keen on knowing the burden of human brucellosis among abattoir workers in Abuja.

The main objective of this study was to determine the seroprevalence and exposure factors associated with human brucellosis in abattoir workers in Abuja, Nigeria so as to provide baseline information as well as give first indications about the extent of the problem in the study area.

## Methods

**Study sites:** we selected two abattoirs with the largest population of abattoir workers and highest daily slaughter of food animals on the basis of the information provided by the FCT Department of Agriculture and Rural Development as study areas out of the five abattoirs across the FCT. Karu abattoir which is government owned and Dei-dei abattoir which is privately-owned were selected for the study.

**Study design:** we carried out a cross-sectional study between December 2010 and August 2011 to determine the seroprevalence of Brucella abortus and Brucella melitensis antibodies in sera of abattoir workers and also to determine the exposure factors associated with seropositivity against Brucella in abattoir workers.

**Study population:** the study population was made up of abattoir workers working at Karu and Dei-dei abattoirs between December 2010 and August 2011.

**Inclusion criteria:** All abattoir workers actively participating in abattoir operations, who were 18 years and above and present at the abattoir at time of visit were included in the study.

**Exclusion criteria:** All meat buyers and children at the abattoir at the time of visit that are not abattoir workers were excluded from the study.

A sero-positive individual was an abattoir worker who on screening for the presence of *B melitensis* or *B abortus* antibodies had a positive brucellosis serological result either by Rose Bengal Plate Test (RBPT) or Enzyme-linked Immunosorbent Assay (ELISA). A sero-negative individual was any person working in the same abattoir whose serum was collected at the same time with the sero-positive individuals and who on screening had a negative brucellosis serological result either by RBPT or ELISA.

### Sampling method and recruitment

We used stratified sampling to select the study subjects. We divided the abattoir workers into six groups based on the nature of their job: butchers; meat sellers; livestock farmers/ traders; abattoir cleaners; veterinarians/ para-veterinarians and others who included security guards, revenue officers and abattoir managers. Butchers were those responsible for slaughtering the animals. Meat sellers were those who sold the meat; Livestock farmers/ traders were those who raised livestock for sale at the abattoir. Veterinarians were professionals who conducted meat inspection after slaughter and para – veterinarians were technicians who provided assistance to the veterinarian. We prepared sampling frames for the different groups from the list of abattoir workers present at time of study and from each frame we selected individuals by simple random sampling.

### Number of subjects and justification of sample size

The sample size used for abattoir workers was determined based on the average number of abattoir workers at the time of this study. Sample size was calculated using the following formula [13] based on total population of workers with an expected proportion of 5% [14] and a margin of error of 3% using a 95% confidence level (CI).

n = Z^2^ x pq/d^2^

Using the above formula, where Z = 1.96, P = 5% or 0.05, q = 1 – p = 0.95 and d = 0.03 A 10% non- response rate was added giving a total sample size of 223. This was the minimum sample size calculated although the actual sample size eventually used for this study was 224 abattoir workers for ease of allocation into strata.

### Method of allocation to study groups

We selected the abattoir workers from each stratum using a proportion of 0.7on the basis of the sample size calculated. A total of 98/140 butchers, 46/66 abattoir cleaners, 43/61 meat sellers, 16/23 veterinarians/ para-veterinarians, 11/16 livestock farmers / traders, 10/14 other workers were selected.

### Laboratory analyses

**Collection of serum samples from abattoir workers:** Three milliliters (ml) of venous blood was collected by qualified medical personnel under very strict hygienic conditions using sterile syringes and needles from the individual abattoir workers into 5ml plain serum tubes and kept in slanted position on ice. Before blood collection, the individual had to give an informed consent to participate in the study. Two medical personnel were trained to administer the questionnaires used for data collection. Clear sera were collected in sterile vials, labeled and stored in a freezer at – 20°C until analysis.

**Serological testing of abattoir workers:** Each serum sample was labeled with a code that corresponded to the study site and subject identification and each was screened for *Brucella abortus* and *Brucella melitensis* antibodies using RBPT specific for each. The tests were done using the standard protocol available in the 2009 Terrestrial Manual [15]. Briefly described, serum samples and antigen were brought to room temperature (22 ± 4°C). Approximately 25µl of each serum was placed on a white tile and an equal volume of antigen was placed near each serum spot. Both were mixed thoroughly using a clean wooden rod and read for agglutination immediately after a 4-minute period. The agglutination reactions were recorded as positive (+) or negative (-) depending on whether there was agglutinations or not. Further screening of sera from abattoir workers were carried out using human IgG and IgM ELISA kits specific for *B melitensis* and the results were correlated with those of the RBPT test done earlier. The reagents in the kit were reconstituted and the test procedure was carried out according to manufacturers’ instructions. Individuals were considered as positive based on a positive RBPT or ELISA result.

**Pre-testing of data collection tool:** we pre-tested our questionnaire at the Jos abattoir which is located in Plateau State, Nigeria and in the same geo-political zone as Abuja and this led to the modification of our data collection instrument.

**Data collection:** data for the study were collected through interviewer-administered questionnaires by well-trained medical personnel at the time of sample collection from the abattoir workers. All abattoir workers meeting eligibility criteria were interviewed.

### Statistical analyses

Data collected were entered, cleaned and analyzed using Epi-info version 3.3.2 software. We used descriptive and analytical statistics to summarize the data obtained. We compared the differences in sero-positives and sero-negatives across the investigated variables using odds ratio and chi-square tests. Test results were considered as significant if the chi-square test p-value was < 0.05. Factors that were found to be significant in the bivariate analyses were subjected to multivariate analyses using a logistic regression model to control for confounding and test for effect modification. In the model used for multivariate analysis, we included all the factors that were biologically plausible and significant at p-value < 0.05 in the bivariate analysis. By stepwise elimination we determined factors that remained significantly associated with a positive serological test for brucellosis.

**Ethical considerations:** approval for this study was obtained from the FCT Research Ethics Committee. Permission was also obtained from the management of each abattoir where the study was carried out. Informed consent was obtained from each eligible abattoir worker before questionnaire administration and sample collection.

## Results

Of the 224 abattoir workers we screened, 172 (76.8%) were male, and their ages ranged from 18 to 67 years. The mean age of study participants was 30years with a standard deviation (SD) of nine years. Thirty-eight (17.0%) of the individuals screened were positive for human brucellosis using RBPT. Twenty-two (9.8%) of the workers were positive for brucellosis with human IgG ELISA test and 18(8.0%) were positive with human IgM ELISA test. A total of 54 individuals tested positive to at least one of the tests giving an overall seroprevalence of 24.1%. Of the 224 abattoir workers screened, 40 (17.9%) had primary education while 68 (30.4%) had secondary education. Of the 54 workers who tested positive to RBPT or ELISA, the majority (87%) were male, over 75% of them were married and more than one half of them resided in rural areas. There appeared to be an increasing trend in the number of individuals who tested positive to RBPT or ELISA as the duration of work at abattoir increased except for those who had worked for over 20years. Abattoir workers who had lower levels of education were more seropositive to human brucellosis comparatively (Table 1).

**Table 1:** Demographic Characteristics of Seropositive and Seronegative Abattoirs workers in Abuja, 2011

Characteristics	Seropositive Individuals N = 54%		Seronegative Individuals N =170%	
**Sex**				
Male	47	87.0	125	73.5
**Marital Status**				
Married	43	79.6	115	67.6
**Residence**				
Rural	29	53.7	78	45.9
**Duration of work**				
< 1 year	1	1.9	12	7.1
1 – 5 years	10	18.5	51	30.0
6 – 10 years	15	27.8	63	37.1
11 – 20 years	22	40.7	34	20.0
>20 years	6	11.1	10	5.9
**Educational Level**				
None	7	13.0	32	18.8
Arabic/Islamic school	12	22.2	46	27.1
Primary	18	33.3	22	12.9
Secondary	17	31.5	51	30.0
Tertiary	0	0.0	19	11.2

**Figure 1:**
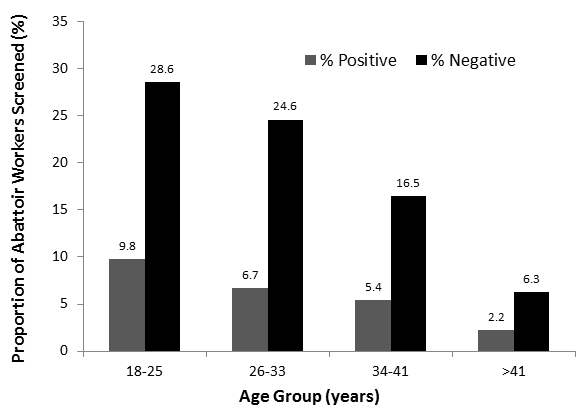
Age distribution of seronegative and seropositive Abattoir Workers screened in Karu and Dei-dei abattoirs in Abuja, 2011

**Figure 2:**
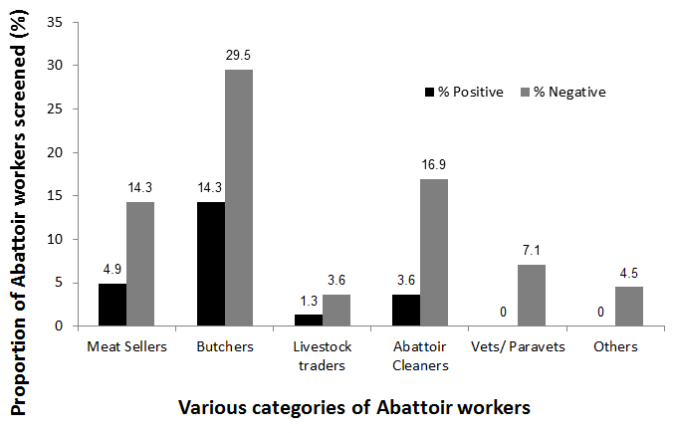
Distribution of seronegative and seropositive individuals among the various groups of Abattoir Workers in Abuja, 2011

The majority of the abattoir workers tested was within the ages 18-25 years; this age group also had more individuals who tested positive to the brucellosis tests. The age group with the least affected individuals was that of above 41 years of age (Figure 1). Seroprevalence varied significantly amongst the various occupational groups working at the abattoirs in Abuja. Among the 54 workers who tested positive to at least one of the tests, butchers (59.2%), meat sellers (20.4%) and abattoir cleaners (14.8%) were the majority. Seroprevalence was highest among butchers (32.7%) whose main job was slaughtering of animals (Figure 2).

Handling aborted fetuses was significantly associated with *Brucella* seropositivity (p = 0.04) (Table 2). Male workers were significantly more likely to be seropositive to human brucellosis (p = 0.04). Abattoir workers who were involved in slaughtering of animals were more likely to be seropositive to human brucellosis (p = 0.02) especially when they sustained an injury (p = 0.02) as well as among those who consumed raw meat while working (p = 0.02). Individuals who had worked in the abattoir for over 5 years were more likely to be seropositive to human brucellosis (p = 0.04). Workers who were involved in milking animals were also more likely to be seropositive to human brucellosis (p = 0.04) (Table 2).

**Table 2:** Bivariate Analysis of Factors Associated with Human brucellosis in Abattoir Workers in Abuja, 2011

Variable	Seropositive N (%)	Seronegative N (%)	OR (95% CI)	P value
**Work Exposure**				
Handling aborted foetus	29 (53.7)	65 (38.2)	1.87 (1.01 – 3.48)	0.04*
Slaughtering Animals	35 (64.8)	77 (45.3)	2.22 (1.18 – 4.20)	0.02*
Milking Animals	8 (14.8)	9 (5.3)	3.11 (1.14 – 8.52)	0.04*
Slaughtering Animals with a cut	34 (63.0)	75 (44.1)	2.15 (1.15 – 4.04)	0.02*
Working as a Butcher	32 (59.3)	66 (38.8)	2.29 (1.23 – 4.28)	0.01*
Working at abattoir for over five years	43 (79.6)	107 (62.9)	2.30 (1.11 – 4.78)	0.04*
Being a male worker	47 (87.0)	125 (73.5)	2.42 (1.20 – 5.74)	0.04*
Selling Meat	18 (33.3)	47 (27.6)	1.31 (0.68 – 2.53)	0.52
Not wearing gloves	52 (96.3)	150 (88.2)	3.47 (0.78 – 15.34)	0.14
**Food Exposure**				
Eating while working	38 (70.4)	120 (70.6)	0.99 (0.51 – 1.94)	0.89
Eating raw meat	12 (22.2)	16 (9.4)	2.75 (1.21 – 6.26)	0.02*
Drinking unpasteurized milk	39 (72.2)	125 (73.5)	0.94 (0.47 – 1.86)	0.99
Drinking milk products made from raw milk	51 (94.4)	150 (88.2)	2.27 (0.65 – 7.95)	0.29
Process milk products	11 (20.4)	21 (12.4)	1.82 (0.81 – 4.06)	0.21

* Values significant at p < 0.05

In the logistic regression model used, the following factors that were significant at bivariate analysis at a p < 0.05 handling aborted fetuses, slaughtering animals, milking animals, slaughtering animal when butcher has an injury, working as a butcher, working at the abattoir for over 5 years and eating raw meat, were included in the model. After controlling for age and using a stepwise elimination approach, only two factors remained statistically significant in the logistic regression model. In our final logistic regression model, consuming raw meat while working and working in the abattoir for over 5 years remained associated with seropositivity to Human brucellosis (Adjusted Odds Ratio (AOR) = 2.64; CI 95% = 1.14-6.14, p = 0.02) for those who ate raw meat and (AOR = 2.47; CI95% = 1.15 – 5.30, p = 0.02) for those who had worked at the abattoir for over 5 years respectively. However there was no association found between seropositivity to human brucellosis and other variables investigated (Table 3).

**Table 3:** Factors found to be significantly associated with Human brucellosis among Abattoir Workers in Abuja in 2011 in a Logistic Regression Model

Variables	Odds Ratio	95% CI	P value
**Work Exposure Factors**			
Handling Aborted Fetuses	1.87	1.01 – 3.48	0.04
Slaughtering Animals	2.22	1.18 – 4.20	0.02
Milking Animals	3.11	1.14 – 8.52	0.04
Slaughtering Animals with a cut	2.15	1.15 – 4.04	0.02
Working as a Butcher	2.29	1.17 – 4.49	0.01
Working at the Abattoir for over 5 years	2.47	1.15 – 5.30	0.02*
**Food Exposure Factors**			
Eating Raw Meat while working	2.64	1.14 – 6.14	0.02*

* Values that remained significant in the logistic regression model. In the final logistic regression model used for the multivariate analysis, the age group of the abattoir workers was included.

## Discussion

Our findings show that the seroprevalence of human brucellosis among abattoir workers was high in the two busiest abattoirs in Abuja. Among the various categories of abattoir workers that we screened, butchers had the highest seropositivity rate. Factors associated with seropositivity for brucellosis were handling aborted foetuses, slaughtering animals when the butcher has an injury, consuming raw meat, occupational exposure of over 5 years and milking animals. In addition, males appeared to be more at risk of infection with brucellosis. However, it should be noted that butchering is a male-dominated activity and this may have accounted for this finding. Workers with over 20 years of service in the abattoir seem to be less at risk of infection. This category of age of service will usually have become senior butchers with many apprentices working under them and are rarely responsible for the direct slaughtering but usually serve in supervisory or advisory capacities.

This study highlighted that among the various occupational groups in the abattoir, seroprevalence of brucellosis was highest among butchers whose main job was slaughtering of animals, followed by livestock traders, meat sellers and then abattoir cleaners when compared with the rest of the workers. Similar findings have been documented from studies done in South west Nigeria, Tanzania and Egypt [16–18]. Seroprevalence was highest among butchers, suggesting that they are more at risk compared to the rest of the workers probably because of their close contacts with blood and tissues of infected animals.

Veterinarians and para-veterinarians, who are considered to be at high risk because of the nature of their jobs, were not found to be seropositive to the *Brucella*organism in this study (Figure 2). This may be attributed to their use of personal protective equipment (PPE) since they are aware of the zoonotic nature of the *Brucella*organism or due to the short exposure time of veterinarians at the abattoir during meat inspection. This finding is supported by the study of Awad (1998) done in Palestine [19].

We found that the likelihood of developing human brucellosis at the abattoir was associated with handling aborted foetuses and this was statistically significant (p = 0.04). This is consistent with the reports of studies done in Tanzania and Chad indicating that brucellosis in humans was strongly associated with handling aborted foetuses and placenta of infected animals [17, 20, 21].

Human brucellosis can be transmitted by inoculation through cuts and abrasions in the skin [8]. From this study, we found that slaughtering animals especially when the butcher has an injury was associated with acquiring brucellosis among abattoir workers. Other studies have reported similar associations of persons with bruised skin or cuts and infection with *Brucella* [22, 23].

We found that consuming raw meat was associated with acquiring human brucellosis in abattoir workers. This finding is similar to the findings of studies done in Central Sudan where it was reported that eating raw liver or other offal with spices was found to be an important epidemiological factor in contracting brucellosis [24]. A study done in Tanzania reported that eating uncooked meat was a common practice among brucellosis infected abattoir workers [25]. A similar study in Saudi Arabia also reported that eating raw liver was a risk factor for developing brucellosis among households that raised livestock [26]. Consumption habits coupled with close contact with infected animals are factors necessary for the spread of brucellosis in man [27]. It has been documented that people acquire infection through consumption of contaminated raw milk, milk products, blood and undercooked meat [18, 19, 28–31]. Though in our study we found no association between acquiring human brucellosis and drinking unpasteurized milk (p = 0.99) however, we will still emphasize the importance of drinking only pasteurized milk in addition to eating properly cooked meat as the outcome may have been because of the subject group used in this study.

In a study carried out in Pakistan it was reported that a rise in seropositivity to *Brucella* was associated with the duration of occupational exposure with the exception of those with less than 1 year's job duration [32]. This was consistent with findings from this study that acquiring human brucellosis among the workers was associated with working in the abattoir for over 5 years (p = 0.02) hence the probable need for brucellosis screening every 5 years to enhance early detection and possibly reduce the cost of treating complications associated with chronic brucellosis.

According to our findings, seropositivity to human brucellosis was higher among workers who were involved in milking animals (p = 0.04). There are also reports which show that close contacts with animals during milk processing is a risk factor for developing the disease in man [33, 34].

Although a study done in Pakistan, showed that the residence of individuals in rural area was a risk factor for *Brucella* seropositivity in humans [32], our study did not find any association between acquiring human brucellosis and being resident in a rural area (p = 0.32), probably because we did the study in an urban area.

A major limitation of our study was that we excluded abattoir workers who were less than 18 years of age who constitute part of the population of abattoir workers in Abuja. This was because they were not eligible to give informed consent; hence our findings cannot be generalized to the entire population of abattoir workers in Abuja and in the whole of Nigeria. Also since our study was a cross sectional study, causation of brucellosis among abattoir workers cannot be proven. Although it is likely that the abattoir workers were infected in the abattoir, it is also possible that some could have been infected in the rural community where they live especially among livestock where brucellosis is endemic.

## Conclusion

The seroprevalence of human brucellosis among abattoir workers in Abuja was high, with butchers having the highest seropositivity. Slaughtering animals when butcher has an injury, handling aborted fetuses, consumption of raw meat, milking animals and occupational exposure of over 5years were important factors associated with seropositivity for brucellosis. Health education on the risks that abattoir workers face from contracting zoonoses and the consistent use of personal protective equipment by abattoir workers will go a very long way in preventing infection in the population at risk. Based on the findings of this study, the following recommendations were made to the relevant authorities. Government at all levels should organize public enlightenment campaigns aimed at highlighting the importance of zoonoses including brucellosis among abattoir workers who should also be encouraged to go for brucellosis screening every 5 years, and abattoir workers should be discouraged from eating raw meat and educated on adherence to safe animal-product handling practices. A repeat of this sero-survey should be considered at regular intervals as a monitoring tool for the prevalence of human brucellosis and the factors associated with seropositivity in abattoirs in Nigeria. We also recommend a joint sensitization of physicians and veterinarians about the need for detailed brucellosis patient and animal information for effective management.
